# Does Green Really Mean Go? Increasing the Fraction of Green Photons Promotes Growth of Tomato but Not Lettuce or Cucumber

**DOI:** 10.3390/plants10040637

**Published:** 2021-03-27

**Authors:** Paul Kusuma, Boston Swan, Bruce Bugbee

**Affiliations:** Crop Physiology Laboratory, Department of Plants, Soils and Climate, Utah State University, Logan, UT 84321, USA; bostonvswan@gmail.com (B.S.); bruce.bugbee@usu.edu (B.B.)

**Keywords:** cryptochrome, phototropins, LEDs, horticulture

## Abstract

The photon flux in the green wavelength region is relatively enriched in shade and the photon flux in the blue region is selectively filtered. In sole source lighting environments, increasing the fraction of blue typically decreases stem elongation and leaf expansion, and smaller leaves reduce photon capture and yield. Photons in the green region reverse these blue reductions through the photoreceptor cryptochrome in *Arabidopsis thaliana,* but studies in other species have not consistently shown the benefits of photons in the green region on leaf expansion and growth. Spectral effects can interact with total photon flux. Here, we report the effect of the fraction of photons in the blue (10 to 30%) and green (0 to 50%) regions at photosynthetic photon flux densities of 200 and 500 µmol m^−2^ s^−1^ in lettuce, cucumber and tomato. As expected, increasing the fraction of photons in the blue region consistently decreased leaf area and dry mass. By contrast, large changes in the fraction of photons in the green region had minimal effects on leaf area and dry mass in lettuce and cucumber. Photons in the green region were more potent at a lower fraction of photons in the blue region. Photons in the green region increased stem and petiole length in cucumber and tomato, which is a classic shade avoidance response. These results suggest that high-light crop species might respond to the fraction of photons in the green region with either shade tolerance (leaf expansion) or shade avoidance (stem elongation).

## 1. Introduction

In naturally shaded environments, irradiance/photon fluxes in the blue (400 to 500 nm) and red (600 to 700 nm) regions are relatively reduced while the fluxes of photons in the green (500 to 600 nm) and far-red (700 to 750 nm) regions are relatively enriched. In the photobiology literature, the term light is often used to refer to the photon flux, as in blue light, but this terminology does not describe the discrete nature of photons, which drive photobiological reactions. Additionally, light is closely connected to brightness in human perception of photons, thus photon is a preferable term. Here, the terms blue, green, red and far-red photons refer to photons in the regions that induce blue, green, red or far-red color perception.

Plant developmental responses to a relative increase in far-red have been well studied [1,2], with species specific increases in leaf area or stem length empirically described as shade tolerance or shade avoidance. It should be noted that shade tolerance does not often specify an increase in leaf area; instead, morphological changes are discussed in terms of an increase in specific leaf area, which is leaf area divided by leaf mass [3].

Elevated far-red commonly increases leaf area in controlled environments and this response is beneficial because it increases photon capture [4]. Increases in stem length in controlled environments are typically considered detrimental. In contrast to the well-characterized responses to far-red, shade-responses to green photons are less well studied.

Early light-emitting diode (LED) fixtures for horticultural applications supplied only blue and red photons. One of the first studies that investigated the effects of adding green to this type of spectrum found that increasing the fraction of green photons from zero to 24% increased leaf area in *Lactuca sativa* cv. “Waldmann’s Green” by 31% and increased shoot dry mass by 47% [5]. This early finding created a sustained interest in considering green photons to horticultural fixtures in order to promote growth [6,7]. However, more recent studies have shown contradictory results to Kim et al. [5] (e.g., [8]), suggesting the need for a reanalysis of the beneficial effects of green photons on plant growth – especially with continued emerging evidence that green photons act antagonistically against blue photons through the photoreceptor cryptochrome.

Cryptochromes are one of the two most well-studied families of blue photon receptors, and they primarily modulate plant growth through the control of gene expression. The other well studied family of blue photon receptors are phototropins, which primarily modulate plant growth through interactions with membranes [9,10]. Sunlight has a relatively high fraction of blue photons, which cause reduced stem and leaf elongation. Studies investigating hypocotyl elongation in *Arabidopsis thaliana* mutants have indicated that cryptochromes are the primary photoreceptor influencing the decrease in stem length [11,12]. Longer-term studies in pea have corroborated this finding as greenhouse grown plants lacking cryptochrome were 20 to 40% longer than the wild-type plants [13]. Phototropins play an early role in reducing hypocotyl elongation at the onset of blue photons [14], but this rapid response does not appear to have a prolonged effect [12]. The role of these photoreceptors in leaf expansion in mature plants is less well studied.

Studies have typically found that increasing the fraction of blue photons decreases leaf area/plant diameter in the horticultural crops lettuce [8,15,16,17,18,19,20,21,22,23,24], cucumber [25,26,27,28] and tomato [26,29], but these effects are not always statistically significant [17,18,26,28,30,31,32,33,34] and occasionally go in the opposite direction [18]. Leaf area is generally highly correlated with dry mass (yield). Thus, increasing the fraction of blue photons also typically result in decreased dry mass [8,15,16,20,21,22,24,25,26,28,35,36], although this is not always the case [17,18,19,25,26,27,28,29,30,31,32,34,36,37,38,39,40,41,42]. In addition to reducing leaf area and yield, blue photons have also been shown to reduce stem and petiole length in cucumber and tomato [25,26,28,32,33], indicating that the effects of blue photons on manipulating cryptochrome activity in *Arabidopsis* extend to these horticultural species. We review 29 studies spanning 19 years on the effects of blue photon fraction in lettuce, cucumber and tomato in Table S1. Comparisons are complex because the studies were conducted at multiple temperatures, study durations, photon fluxes, photoperiods and cultivars.

Unlike spectral distributions that lack either blue or red photons [25,26,43], growing plants in the absence of green photons does not necessarily induce abnormal morphology [25,26]. Studies from the past two decades have suggested that green photons act antagonistically against blue photons to modulate the action of the photoreceptor cryptochrome in a similar manner to the red and far-red antagonism in the photoreceptor phytochrome. In cryptochrome, the flavin adenine dinucleotide (FAD) chromophore has three potential states. The oxidized form, FADox, is abundant in the dark, and upon photon absorbance it converts into the semi-reduced radical state (flavosemiquinone, FADH^o^), which is the active form. Photon absorbance by FADH^o^ induces conversion into the fully reduced (FADH^–^) state, which is inactive [44,45]. The absorbance spectra of FADox shows a sensitivity to blue photons at about 450 nm, with little absorbance beyond 500 nm, while comparatively the absorbance spectrum of FADH^o^ shows relative lower absorbance in the blue region and higher absorbance in the green region [46]. The fully reduced form has a unique absorbance spectrum [47,48], but there are no models of cryptochrome activity that suggest a molecular change in FADH^–^ by photon absorbance [44,49]. This model indicates that green photons ought to partially inhibit/reverse blue induced decreases in stem elongation. If cryptochrome affects leaf expansion, then green photons may increase leaf area and yield.

This hypothesis has been evaluated in multiple studies. Although increasing the green photon flux can induce shade morphology (e.g., increased petiole / total leaf length and decreased leaf angle) in *Arabidopsis thaliana* [50,51], the effect of green photons in horticultural crops is inconsistent. For example, although some studies in cucumber and tomato showed an increase in stem elongation in response to increasing green fraction [26] many others have shown no response of stem or petiole length to increasing the fraction of green photons [25,26,27,29,33], and occasionally studies show a decrease in stem length [28], which is in the opposite direction than expected.

Additionally, replacing red photons with green photons under a constant fraction of blue has increased leaf area and dry mass in some studies [5,20,22,26,39,41,52], but most studies show no response, and several show an opposite response [8,20,23,25,26,27,28,29,33,36,38,40] (also see Table S2). Overall, the expected morphological (and subsequent growth) responses to blue and green photons do no always occur in the horticultural crops lettuce, cucumber and tomato.

The photosynthetic photon flux density (PPFD), or intensity, affects morphology. One notable example is that leaf thickness typically increases with increasing photon flux. Thick and thin leaves are referred to as sun and shade leaves. This response has recently been partially explained by the involvement of both cryptochromes and phototropins [53]. In some studies, the blue fraction has been found to be better a predictor of stem elongation and leaf expansion, while in other species and other studies, absolute blue intensity has been found to be a better predictor of morphological responses [26,30,54,55]. The extent of interactions between photon quality and quantity is not well studied.

Previous studies have investigated some of the following interactions: (1) the effect of blue photons between 10 and 30% blue [8,26], (2) interactions with green photons at multiple levels of blue [8,20], and (3) interactions with intensity [26]. We sought to investigate all three parameters and their interactions. We hypothesized that (1) increasing the fraction of blue photons would reduce plant size (e.g., leaf area, dry mass and stem length), while increasing the fraction of green photons would increase plant size; (2) the effect of blue photons would be more significant at lower intensities (as this would cause photoreceptors to be under-saturated); and (3) the effect of green photons would be more significant at a lower fraction of blue photons.

## 2. Materials and Methods

### 2.1. Plant Material and Cultural Conditions

Lettuce (*Lactuca sativa*, var. Red Salad Bowl), tomato (*Solanum lycopersicum*, cv. Early Girl) and cucumber (*Cucumis sativa*, var. Boston Pickling) seeds were direct seeded then thinned for uniformity after emergence leaving four plants per module. Planted root modules were randomly placed into the 16 treatment chambers. Each chamber had dimensions of 20 × 23 × 30 (L × W × H, 13800 cm^3^) with gloss white walls. Fans provided an air velocity of 0.5 m s^−1^ at the top of the canopy. The root modules measured 20 × 18 × 13 (4680 cm^3^) and contained a 1:1 ratio of peat and vermiculite by volume with five grams of uniformly mixed Nutricote ® slow-release fertilizer (16-2.6-11.2, N-P-K, type 100). Root modules were watered to 10% excess as needed with dilute fertilizer solution (0.01N-0.001P-0.008K; Scotts ® Peat-lite, 21-5-20; EC = 1 mS cm^−1^), and were allowed to passively drain. Type-E Thermocouples connected to a data logger (CR1000, Campbell Scientific, Logan, UT, USA) continuously monitored ambient air temperature at the top of the plant canopy. Day/night temperature was 23/20 °C, with less than 1 °C variation over time and 1 °C variation among chambers. CO_2_ concentration was continuously monitored and was identical for all treatments and varied over time between 450 and 500 ppm.

### 2.2. Treatments

**Figure 1:**
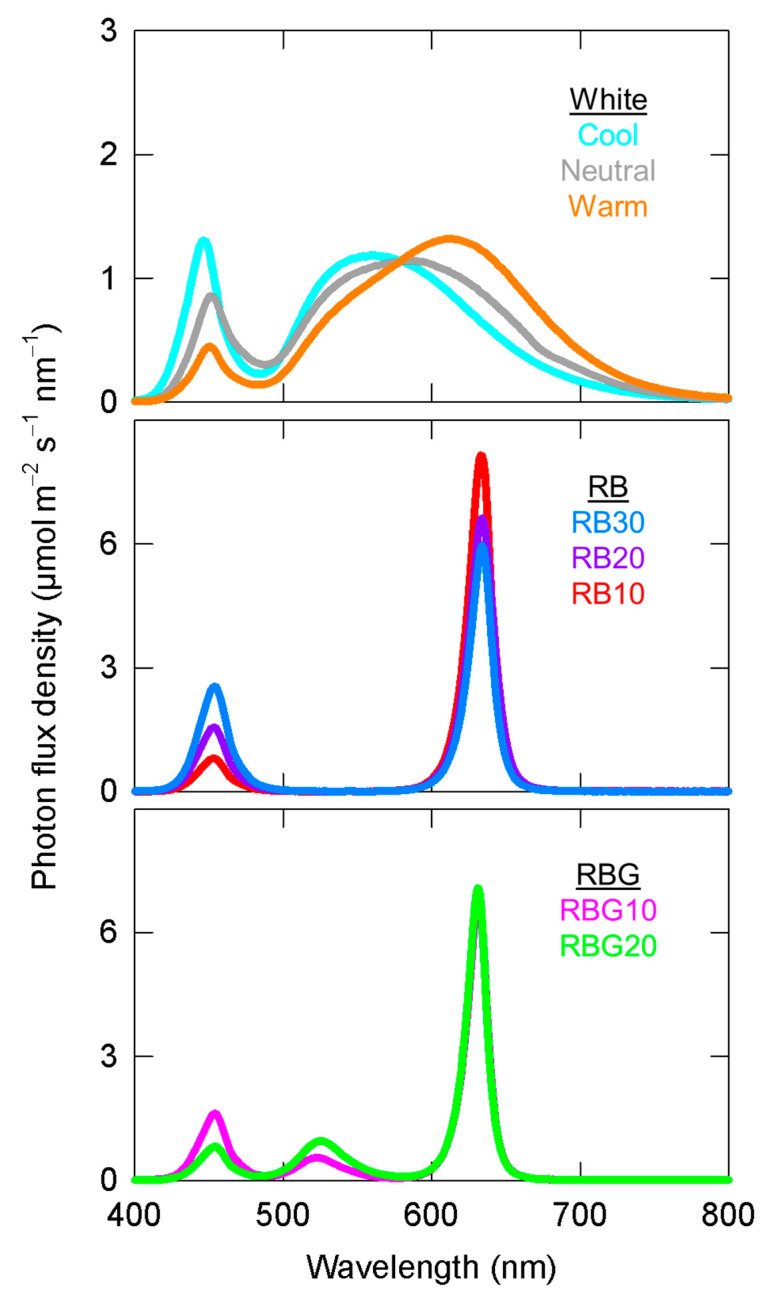
Representative spectral output of each of the eight treatments at a PPFD of 200 µmol m^−2^ s^−1^. (Top) the three white LEDs, (middle) the three RB (red/blue) combinations, (bottom) the two RBG (red/blue/green) treatments.

The system included 16 chambers with eight unique spectral outputs at two intensities for a 16 h photoperiod (PPFD: 200 µmol m^−2^ s^−1^, DLI: 11.5 mol m^−2^ d^−1^; and PPFD: 500 µmol m^−2^ s^−1^ DLI: 28.8 mol m^−2^ d^−1^). Treatments were developed using LEDs (Luxeon Rebel Tri-Star LEDs; Quadica Developments Inc., Ontario, Canada) to output three white (cool, neutral and warm), three red/blue (RB) combinations, and two red/blue/green (RBG) combinations. The RB combination had about 10, 20 and 30% blue, and the RBG treatments contained about 10 and 20% B with 20 or 10% G, respectively. The spectral distributions of the treatments were measured before each replicate study with a spectroradiometer (model PS-200; Apogee Instruments, Logan, UT, USA) and are shown in Figure 1. Blue, green and red as a percentage of the PPFD were calculated for each species at the higher and lower PPFD. These are averaged together in Table 1. PPFD was measured with a full-spectrum quantum sensor (MQ-200, Apogee Instruments, Logan, UT, USA) at the top of the plant canopy, and each chamber was adjusted to maintain PPFD at ± 5%.

### 2.3. Plant Measurements

All species were harvested after canopy closure – when the leaves of the four plants in one of the treatments began to touch. This occurred 21 days after emergence in lettuce, 12, 13 and 20 days after emergence in tomato, and 11 or 13 days after emergence in cucumber. At harvest, stem and longest petiole length of the each of the four plants per chamber were measured in tomato and cucumber. Leaf area was measured using a leaf area meter (LI-3000; LI-COR, Lincoln, NE, USA). Leaf area index (LAI, m^2^_leaf_ m^−2^_ground_) was calculated by dividing total leaf area per chamber by the ground area of the chamber. Shoot dry mass (DM) was measured after the tissue was dried at 80 °C for 48 h. Dry mass per unit area (g DM m^−2^_ground_) was calculated by dividing total dry mass by the chamber area. Specific leaf mass (SLM, kg DM_leaf_ m^−2^_leaf_) was calculated by dividing the total leaf dry mass of the four plants by the total leaf area of the four plants. The average stem and longest petiole length from each chamber were used for statistical analysis (four measurements averaged together).

### 2.4. Statistical Analysis

The study was replicated three times (each with four plants per replicate in time). All data were analyzed using R statistical software (R Foundation for Statistical Computing; Vienna, Austria). Blue, green and PPFD effects on the growth parameters in lettuce, cucumber and tomato were determined using lmer and Anova functions with an F statistic. We present significance at *p* < 0.05 (marked with a *). In a mixed effects linear model, percent blue and percent green were treated as continuous variables while intensity was treated as a fixed effect. Replicates were treated as random factors. Interaction terms between these three factors were included in the linear model. The three-way interaction was insignificant for all parameters and was therefore pooled into the error term.

In order to understand the interactions, the effect of blue photons was also analyzed by separating the data by intensity (200 and 500 µmol m^−2^ s^−1^). This separation was also done in the analysis of the effects of green photons, and because green photons have been implicated in the reversal of blue photon effects, the data were further separated for 10 or 20% blue photons. The RB30 treatment was not included in this analysis, and both the cool and neutral white LEDs were considered about 20% blue.

## 3. Results

**Figure 2:**
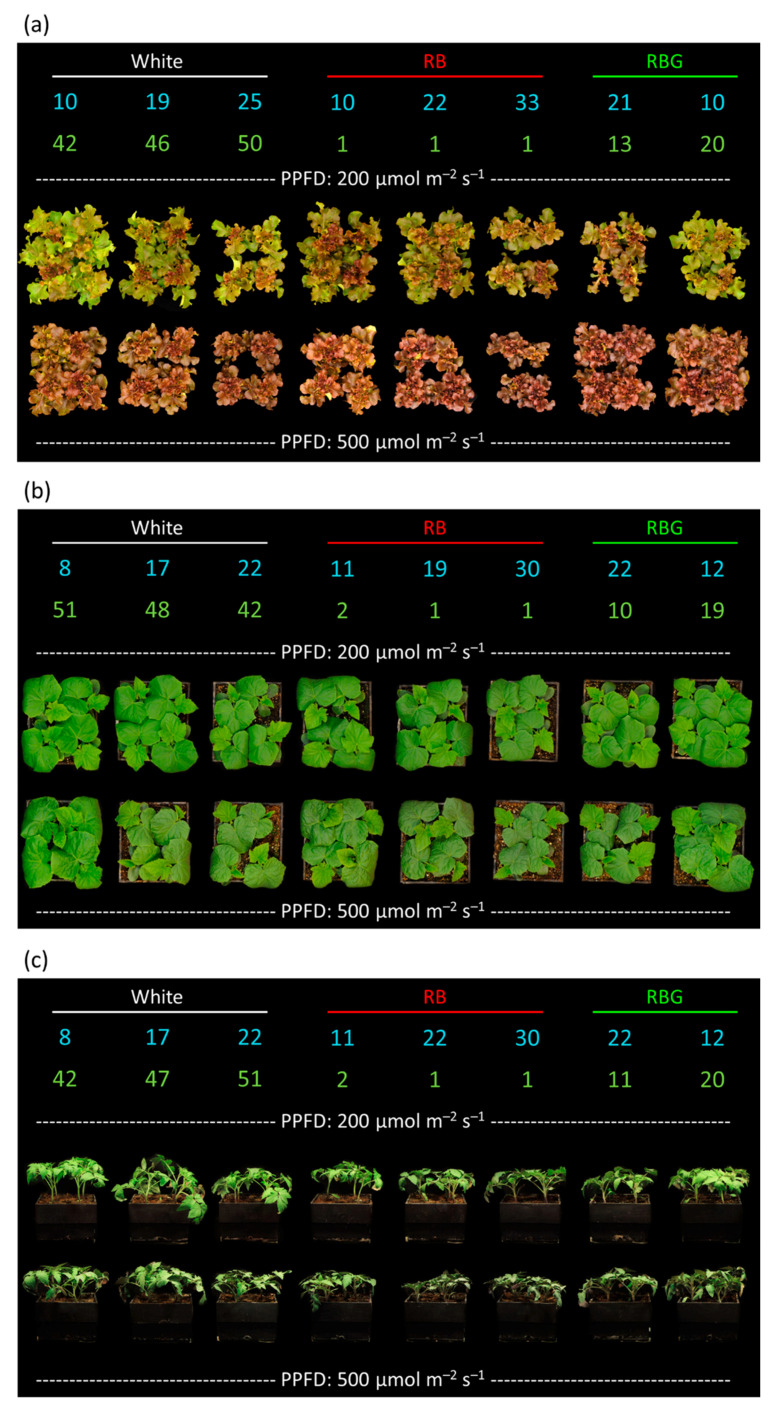
Representative photos from a single replicate of each treatment for (**a**) lettuce, (**b**) cucumber and (**c**) tomato. The average percentages of blue and green are shown above the plants in their respective color. RB: rad/blue; RBG: red/blue/green; PPFD: photosynthetic photon flux density.

Representative photos of the three species in each treatment are shown with the percentages of blue and green in Figure 2.

**Figure 3:**
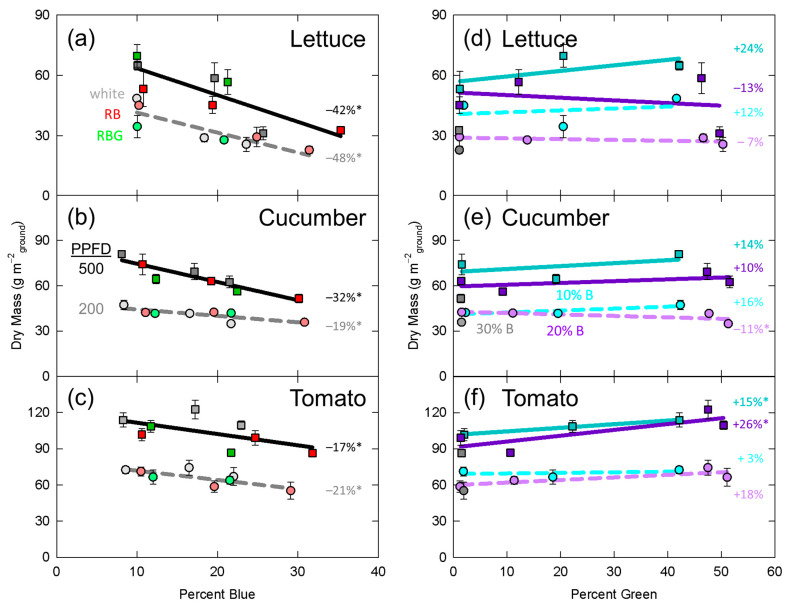
(**a–c**) Effect of percent blue on total dry mass in (**a**) lettuce, (**b**) cucumber, and (**c**) tomato. Squares and the solid black line represent the high intensity treatments (PPFD = 500 µmol m^−2^ s^−1^) and circles and the dashed grey line represent the low intensity treatments (PPFD = 200 µmol m^−2^ s^−1^). Red data points represent RB treatments, green points are RBG treatments and grey points are white LED treatments. The values in percent indicate the change from 10 to 30% blue. (**d–f**) Effect of percent green on dry mass in (**d**) lettuce, (**e**) cucumber, and (**c**) tomato. Solid lines and squares represent the high intensity treatments while the dashed lines and circles represent the low intensity treatment. The cyan data represent treatments with about 10% blue (10% B) and the purple data represent treatments with about 20% blue (20% B). The values in percent indicate the change from zero to 50% green. * represents a significant effect of percent blue or percent green at the *p* < 0.05 level. Error bars represent normalized standard error of n = 3 replicates. RB: rad/blue; RBG: red/blue/green. PPFD: photosynthetic photon flux density.

**Figure 4:**
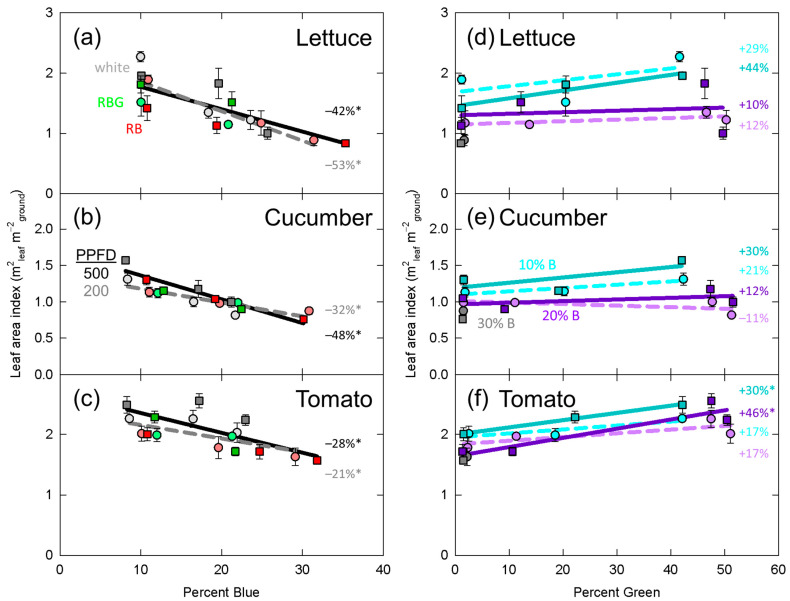
Effect of percent blue on leaf area index in (**a**) lettuce, (**b**) cucumber, and (**c**) tomato; and effect of percent green on leaf area index in (**d**) lettuce, (**e**) cucumber, and (**f**) tomato. * represents a significant effect of percent blue or percent green at the *p* < 0.05 level. See Figure 3 for the meaning of specific labels. Error bars represent normalized standard error of n = 3 replicates.

**Figure 5:**
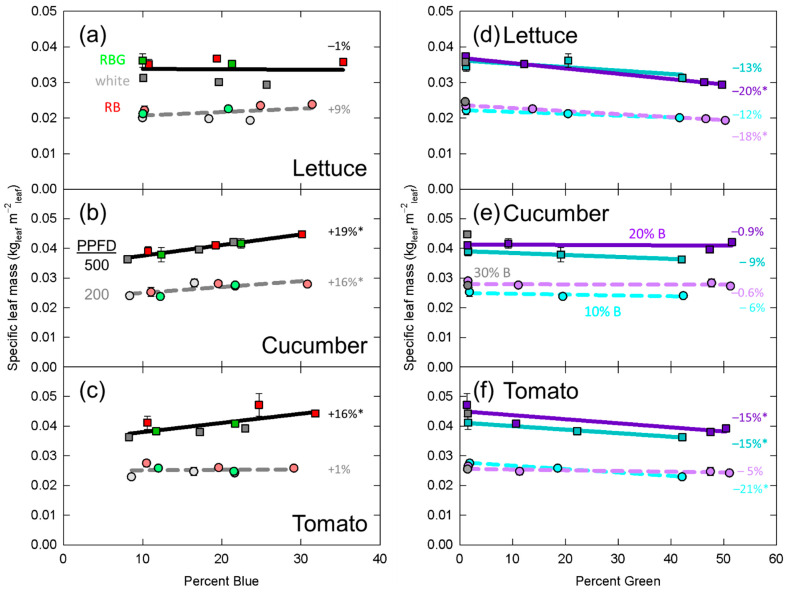
Effect of percent blue on specific leaf mass in (**a**) lettuce, (**b**) cucumber, and (**c**) tomato; and effect of percent green on specific leaf mass in (**d**) lettuce, (**e**) cucumber, and (**f**) tomato. * represents a significant effect of percent blue or percent green at the *p* < 0.05 level. See Figure 3 for the meaning of specific labels. Error bars represent normalized standard error of n = 3 replicates.

**Figure 6:**
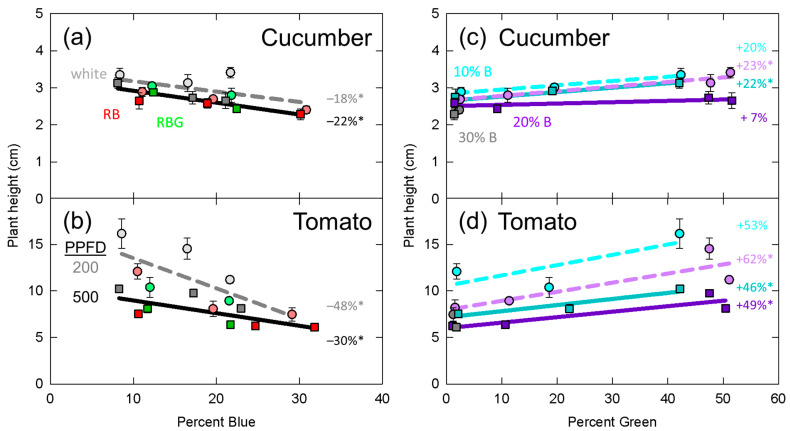
Effect of percent blue on plant height in (**a**) cucumber and (**b**) tomato; and effect of green photons on plant height in (**c**) cucumber and (**d**) tomato. * represents a significant effect of percent blue or percent green at the *p* < 0.05 level. See Figure 3 for the meaning of specific labels. Error bars represent normalized standard error of n = 3 replicates.

**Figure 7:**
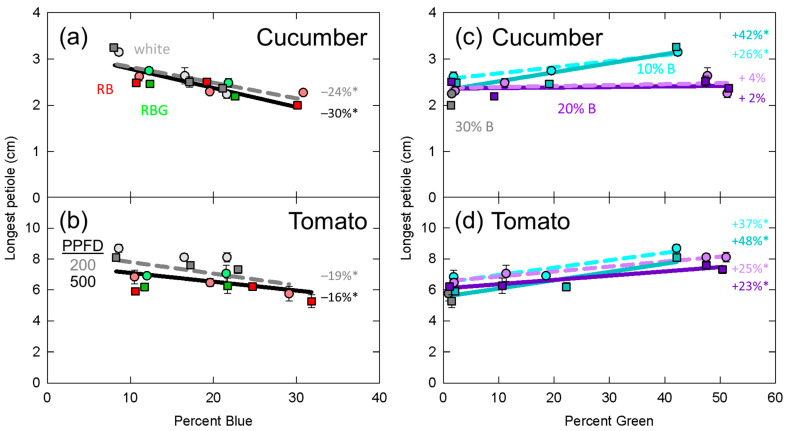
Effect of percent blue on longest petiole length in (**a**) cucumber and (**b**) tomato; and effect of percent green on longest petiole length in (**c**) cucumber and (**d**) tomato. * represents a significant effect of percent blue or percent green at the *p* < 0.05 level. See Figure 3 for the meaning of specific labels. Error bars represent normalized standard error of n = 3 replicates.

Significant effects from the mixed effects linear model are presented in Table 2. In Figure 3, Figure 4, Figure 5, Figure 6 and Figure 7, data for dry mass, LAI, SLM, plant height and longest petiole length for each replicate were normalized to the grand mean of the three replicates and standard error bars represent the normalized error. The resulting percent change between 10 and 30% blue or zero and 50% green is shown. These graphs show the significance of the separated data (e.g., the effect of increasing green photons at 10% blue and a PPFD of 200 µmol m^−2^ s^−1^).

### 3.1. Dry Mass

The higher PPFD (500 µmol m^−2^ s^−1^) resulted in an increased dry mass in all three species (Table 2, Figure 3). Dry mass significantly decreased with increasing percent blue in all three species (Table 2, Figure 3a–c). In cucumber, percent blue interacted with intensity, indicating that the slope of the linear model was significantly different at both intensities (Table 2). Following this interaction, increasing the percent blue from 10 to 30% decreased cucumber dry mass by 32% at the higher PPFD, but only decreased it by 19% at the lower PPFD (Figure 3b).

Percent green significant increased dry mass in tomato, but had no effect in lettuce or cucumber (Table 2, Figure 3d–f) and there was no interaction with PPFD for any species. There was an interaction between percent blue and percent green photons in lettuce, where dry mass trended upward with an increasing fraction of green photons with 10% blue, but trended downward at 20% blue (Figure 3d).

### 3.2. Leaf Area Index

Increasing the fraction of blue photons decreased LAI in all three species (Table 2, Figure 4a–c). PPFD had a significant effect on LAI in cucumber, but not lettuce or tomato. On average, LAI in cucumber was 7% higher in the high PPFD treatment compared to the low PPFD treatment, indicating that this significant effect is not biologically important. Similar to dry mass, there was a significant interaction between percent blue and PPFD in cucumber (Table 2). At the higher PPFD, increasing percent blue from 10 to 30% decreased LAI by 48%, but at the lower PPFD LAI was only decreased by 32% (Figure 4e).

Increasing the fraction of green photons increased LAI in tomato, with no significant effects in lettuce or cucumber (Table 2, Figure 4d–f). There was an interaction between percent blue and percent green in lettuce and cucumber but not tomato. This interaction can be observed in the separated data, where percent green appears to have an effect at 10% blue, but not 20%, although in lettuce this was only statistically significant at the higher intensity (Figure 4d,e). In cucumber, there was a significant interaction between percent green and PPFD. The separated cucumber data show that LAI trended upward more at the higher PPFD than the lower PPFD (Figure 4e).

### 3.3. Specific Leaf Mass

Specific leaf mass is an indicator of leaf thickness; as SLM increases, leaf thickness increases. Increasing the percent blue photons increased SLM in cucumber and tomato, but had no effect on SLM in lettuce (Table 2, Figure 5b,d). Additionally, there was a significant interaction between PPFD and percent blue in tomato (Table 2). When the data were separated for intensity this interaction in tomato appeared to be explained by a significant effect of percent blue at the higher PPFD, but no effect at the low PPFD (Figure 5c). In all cases, SLM was higher at a PPFD of 500 µmol m^−2^ s^−1^ compared to a PPFD of 200 µmol m^−2^ s^−1^.

Increasing the fraction of green photons decreased SLM in lettuce and tomato, but had no effect on cucumber (Table 2, Figure 5d,f). In lettuce, percent green interacted with PPFD to predict SLM, but this effect does not appear biologically important (Figure 5d).

### 3.4. Plant Height

Increasing the fraction of blue photons significantly decreased plant height in both cucumber and tomato (Table 2, Figure 6a,b). The higher PPFD resulted in reduced plant height in both species. Additionally, there was an interaction between percent blue and PPFD in tomato (Table 2). This interaction is apparent in Figure 6b, where plant height decreased by 48% at the lower PPFD, but only decreased by 30% at the higher PPFD.

Plant height significantly increased with increasing green photon fraction in both tomato and cucumber (Table 2, Figure 6c,d). These effects were more dramatic in tomato compared to cucumber, with a 50% increase in stem length as percent green increased from zero to 50% in tomato, but only about 20% increase in height in cucumber. Additionally, in tomato, there was a significant interaction between percent green and percent blue (Figure 6d).

### 3.5. Longest Petiole Length

The results for longest petiole length in cucumber and tomato followed a similar trend to plant height with significant decreases as percent blue increased (Table 2, Figure 7a,b). Additionally, there was a significant effect of PPFD in tomato.

Similar to plant height, increasing the fraction of green photons significantly increased the petiole length in both cucumber and tomato (Table 2, Figure 7c,d), and there was a significant interaction between blue and green fraction in cucumber. This interaction can be seen in Figure 7c where increasing percent green increased petiole length by 26 and 42% at 10% blue, but had no effect at 20% blue.

## 4. Discussion

### 4.1. Mechanism Underlying Specific Leaf Mass

At high light intensity, leaf thickness increases due to an increase in both cell elongation and cell division along the abaxial to adaxial axis. This has led to the categorizations of sun and shade leaves [56]. It is therefore unsurprising that SLM was significantly increased at the higher intensity (PPFD = 500 µmol m^−2^ s^−1^) compared to the lower intensity (PPFD = 200 µmol m^−2^ s^−1^) in all three species (Table 2, Figure 5). Previous studies using *Arabidopsis thaliana* mutants deficient in the blue photoreceptors cryptochromes [57] or phototropins [58] showed no difference in leaf thickness from the wild-type when exposed to high or low photon intensity. This led to confusion regarding the mechanism controlling this response, which had long been suspected to be tied to these photoreceptors [59]. Hoshio et al. [53] showed that the increased cell elongation along the abaxial to adaxial axis was absent in the *cry1cry2phot1phot2* quadruple mutant, indicating that all four photoreceptors work together to induce this response at a specific stage of leaf development. They also showed that this quadruple mutant growing under pure blue photons had fewer cell layers than the wild-type, possibly implicating these photoreceptors in the cell division response. They concluded that the photoreceptors only partially explained the response to high intensity, and that other unknown mechanisms remain.

Previous studies have shown that increasing the fraction of blue photons increased SLM, indicating an increase in leaf thickness [20,25,26], but other studies have showed no response [21,22,26]. In this study, increasing the fraction of blue photons increased SLM in both cucumber and tomato (Table 2, Figure 5b,c).

The chromophore in cryptochrome is FAD and the chromophore in phototropin is a flavin mononucleotide (FMN). These two chromophores (FAD and FMN) are structurally very similar, and as such they have similar absorbance properties for each of their oxidized, semi-reduced and fully reduced states [60]. For cryptochrome, FADH^o^ has been implicated as the active state, and absorbance of green photons by this state can convert it into the fully reduced, inactive state [44,45,61]. Despite the similar absorbance properties of FMN compared to FAD, no inhibition of phototropin action by green photons has been described, and intermediate forms of FMN are extremely transient [62]. In a similar manner, although phototropins have been implicated in stomatal opening [63], the reversal of blue photon induced stomatal opening by green photons does not appear to be under the control of phototropins [64]. Nonetheless, SLM appears to be modulated by both of these photoreceptors [53], and thus green photons could reverse the blue induced increase in SLM through cryptochrome.

In this study, both lettuce and tomato showed a significant decrease in SLM with an increasing fraction of green photons (Table 2, Figure 5d,f). This response is in the expected direction if green photons reverses blue-photon-induced increases in SLM. 

Photoreceptors may be saturated at higher photon fluxes, meaning that spectral shifts at higher intensity may have a smaller impact on plant morphology than at lower intensities. By contrast, preferential absorption of blue photons by pigments other than the blue photon receptors may minimize plant responses to lower intensities of blue photons. The latter scenario may be the case for the interaction between percent blue and PPFD in tomato SLM, as increasing percent blue at the higher PPFD increased SLM by 16%, but appeared to have no effect at the lower PPFD (Figure 5c). The interaction between percent green and PPFD in predicting SLM in lettuce is more difficult to explain as the separated data do not show large differences between the intensities (Figure 5d).

Despite these perplexing interactions, the overall response of SLM to an increase in the fraction of blue and green photons is generally consistent with the role of cryptochromes and phototropins.

### 4.2. Leaf Expansion

Mechanisms underlying leaf area are complex due to interactions with photosynthesis. Here, we review the potential role of phototropins and cryptochromes on the control of leaf expansion under blue and green photons.

In a young seedling, blue photons contribute to early cotyledon expansion, and both cryptochromes and phototropins are involved in this response [11,12]. Mature *Arabidopsis thaliana* mutants lacking phototropins have smaller curled leaves compared to wild-type plants [65,66,67]. This is a puzzling considering observed decreases in leaf area as the fraction of blue photons increases (Table S1, Table 2, Figure 4a–c). It is possible that phototropins simply increase cell expansion in all directions, and thus do not contribute to the blue photon induced decreases in leaf expansion.

Cryptochromes have been implicated in low blue shade avoidance responses like hyponasty [68], but the role of cryptochromes in leaf expansion of mature plants is less well determined. Fig. 1a in Wu and Yang [69] showed overexpressing cryptochrome visually decreased plant diameter (leaf expansion and petiole elongation), while mutants lacking cryptochrome looked stretched compared to the wild-type. Overexpression of cryptochrome in rice led to a significant reduction in the expansion of the secondary leaf blade [70]. These results provide some evidence for the role of cryptochrome in reducing leaf expansion upon blue photon perception.

Considering the potential role of cryptochrome and phototropin in SLM, it follows that increasing leaf thickness through cell elongation in the abaxial to adaxial direction may decrease leaf expansion. Dougher and Bugbee [71] found that the blue induced a decrease in soybean leaf area was partially caused by a decrease in epidermal cell area. They also determined that the observed decrease in leaf area was influenced by a decrease in epidermal (anticlinal) cell division (as measured by dividing leaf area by cell area). Yano and Terashima [72] concluded that high intensity photon flux did not change the rate of total cell division, but increased periclinal cell division at the expense of anticlinal cell division. Hoshino et al. [53] also showed that under monochromatic blue photons, pericinal cell division was reduced in the quadruple mutant (*cry1cry2phot1phot2*). Altogether, increasing the fraction of blue photons (perceived by cryptochromes) may both increase periclinal cell division at the expense of anticlinal cell division, and increase cell expansion along the abaxial to adaxial axis at the expense of elongation along the perpendicular axis. 

Although the mechanisms underlying the effect of blue photons on leaf expansion are not clear, cryptochromes appear to modulate the response. It is difficult to determine the role of phototropins in leaf expansion. Further research is warranted.

### 4.3. Potential Contribution of Photosynthesis to Dry Mass Accumulation

The decreases in dry mass as the fraction of blue photons increased were likely caused in part by the lower quantum yield of blue photons compared to green and red photons. McCree [73] showed that in lettuce (cv. Great Lakes and Big Boston), cucumber (cv. Ohio MR-17) and tomato (cv. Floradel) blue photons had a 24, 33 and 30% lower quantum yield than red photons. Green photons had a 16% lower quantum yield than red photons in all three species. As the blue percentage increased from 10 to 30%, dry mass decreased by 42 and 48% in lettuce, 32 and 19% in cucumber, and 17 and 21% in tomato (Figure 3a–c). Although decreased photosynthesis likely contributed to these decreases in dry mass, the effects were also likely caused by reduction in photon capture (decreased LAI).

### 4.4. Comparison to Previous Studies in Horticultural Species

Increasing the fraction of blue photons decreased LAI in all three species, while increasing the fraction of green photons only increased LAI in tomato (Table 2, Figure 4f). As dry mass is highly correlated with leaf area [74], the decreases in LAI likely contributed to the decreases in dry mass. In lettuce, increasing the fraction of blue photons has often been shown to decrease leaf area and dry mass [8,15,16,20,21,22,24,35], although some studies show no response [26,28,37,38,39,40,41,42] (also see Table S1). Our data showed that increasing the blue photon fraction decreased leaf area and dry mass in lettuce. In cucumber, data from previous publications have generally shown a decrease in leaf area and dry mass [25,26,28], and our data confirm these findings. In tomato, the literature has largely shown no response to increasing the fraction of blue [26,31,32,33], but our data show a significant decrease in LAI and dry mass. The interaction between percent blue and PPFD for both dry mass and LAI in cucumber resulted in a greater response at the higher intensity, suggesting that blue photons were preferentially absorbed by other pigments at the lower intensity.

Despite the early findings of Kim et al. [5], who showed beneficial effects of adding green photons to the growth spectrum, subsequent studies have rarely shown beneficial effects [8,16,23,25,26,27,28,29,33,36,38,40] (also see Table S2). Likewise, we found no beneficial effect (increase in dry mass) of adding green photons to lettuce or cucumber, but we did see a beneficial effect for both leaf area and dry mass in tomato (Table 2, Figure 3d–f, Figure 4d–f). Based on our definition of shade tolerance as an increase in leaf area under shade-light, tomato appeared to exhibit shade tolerance in response to green photons, but lettuce and cucumber did not. 

Green photons have been shown to induce shade avoidance responses in *Arabidopsis thaliana*, but the shade avoidance responses of stem and petiole elongation in horticultural species are often absent [25,26,27,29,33]. By contrast, increasing the fraction of blue photons is regularly observed to decreases stem and petiole length in cucumber and tomato [25,26,28,33]. In this study, despite previous findings, we showed that green photons induced shade avoidance responses in the horticultural species cucumber and tomato (Table 2, Figure 6c,d, Figure 7c,d). We also observed the common response of decreased stem and petiole lengths with increasing blue photon fraction. Unlike the interactions discussed previously, the interaction between percent blue and PPFD in predicting tomato plant height may be explained by saturating photoreceptors at higher intensities.

### 4.5. Interaction between Percent Blue and Percent Green

The interactions between blue photons and green photons in predicting leaf area of lettuce and cucumber, and petiole length in cucumber can be explained by the sensitivity of cryptochrome to blue and green photons. Bouly et al. [44] measured cry2 activation and de-activation by its rate of breakdown (cry2 breaks down in its active form). Compared to applying blue photons alone, green photons provided at three times the rate of blue photons, resulted in minimal differences in the concentration of cry2, but when green photons were applied at ten times the rate, there was a significant increase in the concentration of cry2. This indicates that green photons de-activated cry2 (as measured by a decrease in its degradation) only when applied at ten times, and not three times, the rate of blue photons. More recent studies have estimated the photoconversion coefficients (photoconversion weighting factors) for FAD state changes in vivo are 480 m^2^ mol^−1^ for the activation of cry2 by 450 nm blue photons and 30 m^2^ mol^-1^ for de-activation by 560 nm green photons [61]. Photoconversion coefficients are probability functions that estimate the likelihood of photon absorbance by a pigment and subsequent conversion to another form; they are regularly used in phytochrome research (e.g., [75]). The results of Procopio et al. [61] indicate that blue photons have a 16-fold greater ability to change the state of cryptochrome compared to green photons. Therefore, increasing the flux of green at a higher fraction of blue would be expected to have a reduced response compared to a lower fraction of blue. Although this is complicated by the fact that the cryptochrome photocycle involves three states rather than two, and the more reduced states are only converted back to the oxidized state via dark reversion. A better understanding of this photocycle may provide better models for the action of blue and green photons in future studies. Nonetheless, green photons were more potent at increasing leaf expansion in lettuce and cucumber (Figure 4d,e), and the petiole length in cucumber (Figure 7c) at a lower fraction of blue. The response in tomato plant height is more difficult to explain (Figure 6d).

## 5. Conclusions

Green photons only benefitted tomatoes. These data are contrary to the early findings of Kim et al. [5], who found that increased green fraction from fluorescent lamps increased the growth of lettuce compared to LEDs without green. Higher fractions of green photons in horticultural LED fixtures may benefit tomato production, but not lettuce or cucumber production. White LEDs, which provide a high fraction of green photons, are the most cost-effective type of LED due to their use in general lighting [76]. Thus, their use in horticultural LED fixtures will likely continue for economic reasons.

We define shade tolerance as an increase in leaf area in response to shade-like light, and shade avoidance as an increase in stem elongation. Based on these definitions, tomatoes expressed both shade avoidance and shade tolerance in response to an increased fraction of green photons, while cucumber only exhibited shade avoidance.

Blue photons typically decrease leaf area and dry mass. It therefore seems advantageous to include a low fraction of blue photons in a fixture. From our data, the lowest percentage of blue (10%) produced the highest dry mass. We are now studying the minimal amount of blue needed for normal plant growth.

## Figures and Tables

**Table 1:** Representative ratios of blue, green and red fluxes as a percentage of photosynthetic photon flux density (PPFD). Values from the three species and two intensities deviated less than 10% from the average and thus we present average values. RB refers to treatments comprised of red and blue LEDs and RBG refers to treatments comprised of red, blue and green LEDs.

	White			RB			RBG	
	Warm	Neutral	Cool	RB10	RB20	RB30	RBG10	RBG20
% Blue (400 to 499)	9	18	23	11	21	32	22	11
% Green (500 to 599)	42	47	51	1	1	1	11	20
% Red (600 to 699)	49	35	26	88	78	67	67	69

**Table 2:** Significant effects of percent blue (%B), percent green (%G) and PPFD, along with interaction terms on the parameters of dry mass, leaf area index, specific leaf mass, height and longest petiole. * represents a significant effect at *p* < 0.05. NS, not significant. Let: lettuce; Cuc: cucumber; Tom: tomato.

	Dry Mass			Leaf Area			Specific Leaf Mass			Height		Petiole	
	Let	Cuc	Tom	Let	Cuc	Tom	Let	Cuc	Tom	Cuc	Tom	Cuc	Tom
%B	*	*	*	*	*	*	NS	*	*	*	*	*	*
%G	NS	NS	*	NS	NS	*	*	NS	*	*	*	*	*
PPFD	*	*	*	NS	*	NS	*	*	*	*	*	NS	*
%B*%G	*	NS	NS	*	*	NS	NS	NS	NS	NS	*	*	NS
%B*PPFD	NS	*	NS	NS	*	NS	NS	NS	*	NS	*	NS	NS
%G*PPFD	NS	NS	NS	NS	*	NS	*	NS	NS	NS	NS	NS	NS

## Data Availability

Not applicable.

## Supplementary Materials

The following are available online at https://www.mdpi.com/article/10.3390/plants10040637/s1, Figure S1: Photos of lettuce from other replicates, Table S1: Blue Effects, Table S2: Green Effects.
