# Supplementary material for: Does Green Really Mean Go? Increasing the Fraction of Green Photons Promotes Growth of Tomato but Not Lettuce or Cucumber
Source: Plants (Basel). 2021 Mar 27;10(4):637. doi: 10.3390/plants10040637 (PMC8066606; doi:10.3390/plants10040637)

Table S1. Response of lettuce, cucumber and tomato growth (dry mass) and development (leaf area, stem length and petiole length) to blue photons (B). This analysis only includes the effects of increasing the fraction of blue between 5 and 75% blue, meaning that all treatments contain some red (600 to 699 nm) and blue photons. The complete absence of either red or blue photons often induces abnormal growth and development [25,26], likely caused by under-activation of the photoreceptors cryptochromes and phytochormes [43]. In the Days column the number in bold is the number of days in the treatment, and the other number is the number of days between emergence/planting and moving the plants into the treatments.

Blue Effects											
Species	Cultivar	Range of treatments	PPFD ($\mu mol\ m^{-2}\ s^{-1}$)	period (h)	Parameter	Effect	Citation	Comment	CO_2 (ppm)	Temp	Days
Lettuce	Waldmann's Green	8 and 16%	300	18	Dry Mass	NS	Yorio et al. 2001		1200	23	**21**
	Grand Rapids	6 to 26%	200 & 500	16	Leaf Area	NS	Dougher and Bugbee 2001		1000	26/22	4+**18**
					Dry Mass	↑					
	Red Fire	10 and 26%	300	12	Leaf Area	↓	Ohashi-Kaneko et al. 2007		400	20/18	**37**
					Dry Mass	↓					
	Red Cross	16, 23 and 55%	300	16	Dry Mass	NS	Li and Kubota 2009		-	25/20	10+**12**
	Sunmang	13 to 59%	171	12	Leaf Area	↓	Son and Oh 2013	Grand Rapids lettuce at 47% blue did not fit the trend	400	20	18+**28**
					Dry Mass	↓					
	Grand Rapid TBR				Leaf Area	↓					
					Dry Mass	↓					
	Waldmann's Green	11 to 28%	200	16	Leaf Area	-	Cope et al. 2014	Results for leaf area at 200 $\mu mol\ m^{-2}\ s^{-1}$ are difficult to interpret	430	24.5	**21**
					Dry Mass	NS					
			500		Leaf Area	↓					
					Dry Mass	NS					
	Red Fire	15, 19 and 25%	100	16	Leaf Area	↓	Furuyama et al. 2014	At 200 $\mu mol\ m^{-2}\ s^{-1}$ the highest dry mass occurred in the middle treatment (19% B)	1000	23/20	4+11 +**14**
					Dry Mass	NS					
			200		Leaf Area	NS					
					Dry Mass	-					
			300		Leaf Area	↑					
					Dry Mass	↑					
	Jeokchima	10, 20 and 30%	230	16	Leaf Area	↓	Lee et al. 2014	Decrease in leaf area only occurs between 20 and 30% B	800	22/18	**28**
					Dry Mass	NS					
	Ostinata	12 to 36%	150	16	Dry Mass	-	Kong et al. 2015	Results are difficult to interpret. No apparent trend	1200	25/18	**39**
	Sunmang	13 to 34%	173	12	Leaf Area	↓	Son and Oh 2015		400	20	18+**28**
					Dry Mass	↓					
	Grand Rapid TBR				Leaf Area	↓					
					Dry Mass	↓					
	Green Oak Leaf	23, 30 and 45%	135	16	Dry Mass	-	Chen et al. 2016	Results are difficult to interpret. No apparent trend	350	22/18	**35**
	unspecified	8 to 50%	200	16	Leaf Area	↓	Wang et al. 2016		400	24/20	**30**
					Dry Mass	↓					
	Sunmang	8 to 31%	173	12	Leaf Area	↓	Son et al. 2016	Results are difficult to interpret. Appears to trend downward	400	20	18+**28**
					Dry Mass	↓					
	Waldmann's Green	11 to 28%	200	16	Leaf Area	NS	Snowden et al. 2016		430	-	**21**
					Dry Mass	NS					
			500		Leaf Area	NS					
					Dry Mass	NS					
	Green Skirt	10 to 30%	150	16	Plant diameter	↓	Kang et al. 2016		1000	22/17	14+**28**
	Frill ice	20.7, 21.4 and 26.2%	200	14	Dry Mass	-	Yan et al. 2019	At 200 $\mu mol\ m^{-2}\ s^{-1}$ in 14 h, the highest dry mass occurred in the middle treatment (21.4% B)	800	22/18	20+ **20**
				16		NS					
			250	14		NS					
				16		NS					
	Rouxai	7 to 33%	180	20	Plant diameter	↓	Meng et al. 2019		379 to 402	20	**30**
					Fresh/Dry Mass	↓					
	Rex				Plant diameter	↓					
					Fresh/Dry Mass	↓					
	Rex	17 and 50%	180	24	Dry Mass	↓	Meng and Runkle 2019	Not including FR treatments	-	23	3 + **12**
	Cherokee				Dry Mass	↓					

Table S1. Continued

Species	Cultivar	Range of treatments	PPFD (µmol m^{-2} s^{-1})	period (h)	Parameter	Effect	Citation	Comment	CO_2 (ppm)	Temp	Days
Lettuce	Ziwei	14 to 27%	100	14	Dry Mass	NS	Yan et al. 2019b	At 150, 200 and 250 µmol m^{-2} s^{-1}, treatments had significant differences, but the pattern is not always consistent.	800	22	20+ **20**
			150			-					
			200			-					
			250			-					
			300			↓					
	Rouxai	7 to 55%	180	20	Plant diameter	↓	Meng et al. 2020		410	22	**30/33**
					Dry Mass	↓					
	Green Oak Leaf	20 and 50%	200	18	Dry Mass	NS	Spalholz et al. 2020	A higher 80% B showed significant differences in Red Oak Leaf	704	20	**42**
	Red Oak Leaf				Dry Mass	NS					
	Tiberius	10, 25 and 28% B	210	16	Leaf Area	NS	Zou et al. 2020	The highest dry mass and leaf area occurred in the middle treatment	-(400)	23/21	**15**
					Dry Mass	NS					
Cucumber	Cumlaude	10 to 75%	100	18	Leaf Area	↓	Hernandez and Kubota 2016		512	24.5	**17**
					Dry Mass	↓					
					Stem Length	↓					
	Sweet Slice	11 to 28%	200	16	Leaf Area	NS	Snowden et al. 2016		430	-	**21**
					Dry Mass	NS					
					Stem Length	NS					
					Petiole Length	↓					
			500		Leaf Area	↓					
					Dry Mass	↓					
					Stem Length	↓					
					Petiole Length	↓					
	Zhongnong 26	14 to 27% B	200	12	Leaf Area	↓	Song et al. 2017	Results are difficult to interpret. Lowest dry mass and height were in intermediate treatments. Tallest plants occurred at the highest percent blue	500	25	**15**
					Dry Mass	-					
					Stem Length	- (↑)					
	Zhongnong 16	10, 25 and 28% B	230	16	Leaf Area	↓	Zou et al. 2020		-(400)	23/21	**15**
					Dry Mass	↓					
					Stem Length	↓					
Tomato	Komeett	0, 4 and 16% B (supplemental in GH)	56 + GH	18	Leaf Area	NS	Hernandez and Kubota 2012	Supplemental in GH	512	24.5	7+**11**
					Dry Mass	NS					
					Stem Length	NS					
	Early Girl	25 and 50%	160	18	Leaf Area	↓	Wollaeger and Runkle 2014		-	20	**31/32**
					Dry Mass	NS					
					Stem Length	NS					
	Early Girl	6 to 50%	160	18	Leaf Area	NS	Wollaeger and Runkle 2015		-	20	**31 to 33**
					Dry Mass	NS					
					Stem Length	↓					
	Early girl	11 to 28%	200	16	Leaf Area	↓	Snowden et al. 2016		430	-	**21**
					Dry Mass	↓					
					Stem Length	↓					
					Petiole Length	NS					
			500		Leaf Area	↓					
					Dry Mass	↓					
					Stem Length	↓					
					Petiole Length	↓					
	Komeett	10 to 75%	100	18	Leaf Area	NS	Hernandez et al. 2016		509	25	**21**
					Dry Mass	NS					
					Stem Length	↓					
	Qianxi	25 to 75%	300	12	Leaf Area	-	Liu et al. 2018	Significant differences, but no apparent trends	- (400)	28/18	**30**
					Dry Mass	-					

Table S2. Effect of green photons (G) on growth and development of lettuce, cucumber and tomato. The studies included in this summary generally maintained a constant fraction of blue photons (B), while increasing the fraction of green photons (by simultaneously decreasing the fraction of red photons). In the Days column the number in bold is the number of days in the treatment, and the other number is the number of days between emergence/planting and moving the plants into the treatments.

Green Effects											
Species	Cultivar	Range of treatments	PPFD (µmol m^{-2} s^{-1})	period (h)	Parameter	Effect	Citation	Comment	CO_2 (ppm)	Temp	Days
Lettuce	Waldmann's Green	16% B with or without 24% G	150	18	Leaf Area	↑	Kim et al. 2004		1200	21	**28**
					Dry Mass	↑					
	Red Cross	31, 52 and 70% G	300	16	Dry Mass	NS	Li and Kubota 2009		-	25/20	10+**12**
	Ostinata	27% B with 23, 40 or 51% G	150	16	Dry Mass	↑	Kong et al. 2015		1200	25/18	**39**
	Sunmang	14% B with or without 8% G	173	12	Leaf Area	NS	Son and Oh 2015		400	20	18+**28**
					Dry Mass	NS					
		24% B with or without 8% G			Leaf Area	NS					
					Dry Mass	↑					
	Grand Rapid TBR	14% B with or without 8% G			Leaf Area	NS					
					Dry Mass	NS					
		24% B with or without 8% G			Leaf Area	NS					
					Dry Mass	NS					
	Green Oak Leaf	23% B with 30 or 53% G	135	16	Dry Mass	NS/↓	Chen et al. 2016	NS compared to high yellow, ↓compared to high red	350	22/18	**35**
	Sunmang	About 20% B with 7 or 13% G	173	12	Leaf Area	↑	Son et al. 2016	Two 7% G treatments. NS compared to one, ↑ compared to other	400	20	18+**28**
					Dry Mass	NS/↑					
	Waldmann's Green	1.7 to 41% G in a background of 11 to 14% B	200	16	Leaf Area	↓	Snowden et al. 2016		430		**21**
					Dry Mass	NS					
			500		Leaf Area	NS					
					Dry Mass	NS					
	Green Skirt	10% G in a 10, 20 or 30% B background	150	16	Plant diameter	NS	Kang et al. 2016		1000	22/17	14+**28**
	Frill ice	21% B with 34 or 41% G	200	14	Dry Mass	↑	Yan et al. 2019		800	22/18	20+**20**
				16		NS					
			250	14		NS					
				16		NS					
	Ziwei	18.5% B with 32 or 45% G	100	14	Dry Mass	NS	Yan et al. 2019b		800	22	20+**20**
			150			NS					
			200			↓					
			250			↓					
			300			↓					
	Rouxai	12% B with or without 32% G	180	20	Plant diameter	NS	Meng et al. 2020		410	22	**30/33**
					Dry Mass	NS					
		34% B with or without 33% G			Plant diameter	↓					
					Dry Mass	↓					
		55% B with or without 32% G			Plant diameter	NS					
					Dry Mass	↓					
	Tiberius	15% B with 0 or 25% G	150	16	Leaf Area	↑	Li et al. 2020		1000	24/20	**21**
					Dry Mass	NS					
	Tiberius	27% B with 35 or 45% G	210	16	Leaf Area	↓	Zou et al. 2020		-(400)	23/21	**15**
					Dry Mass	↓					

Table S2. Continued

Species	Cultivar	Range of treatments	PPFD (µmol m^{-2} s^{-1})	period (h)	Parameter	Effect	Citation	Comment	CO_2 (ppm)	Temp	Days
Cucumber	Cumlaude	28% G at 20%B	100	18	Leaf Area	NS	Hernandez and Kubota 2016	No departure from the trend set by B effects	512	24.5	**17**
					Dry Mass	NS					
					Stem Length	NS					
	Sweet Slice	13% B with 2 to 41% G	200	16	Leaf Area	NS	Snowden et al. 2016		430	-	**21**
					Dry Mass	NS					
					Stem Length	NS					
					Petiole Length	NS					
			500		Leaf Area	↑					
					Dry Mass	NS					
					Stem Length	NS					
					Petiole Length	NS					
	Zhongnong 26	20% B with 1 to 44% G	200	12	Leaf Area	NS	Song et al. 2017		500	25	**15**
					Dry Mass	↓					
					Stem Length	NS					
	Zhongnong 16	27% B with 35 or 45% G	230	16	Leaf Area	↓	Zou et al. 2020		-(400)	23/21	**15**
					Dry Mass	↓					
					Stem Length	↓					
Tomato	Early Girl	0 and 25% G as B decreases from 50 to 25%	160	18	Leaf Area	NS	Wollaeger and Runkle 2014		-	20	**31/32**
					Dry Mass	NS					
					Stem Length	NS					
	Early girl	1.7 to 41% G in a background of 11 to 14% B	200	16	Leaf Area	NS	Snowden et al. 2016		430	-	**21**
					Dry Mass	NS					
					Stem Length	NS					
					Petiole Length	NS					
			500		Leaf Area	NS					
					Dry Mass	NS					
					Stem Length	↑					
					Petiole Length	NS					
	Komeett	28% G at 20% B	100	18	Leaf Area	NS	Hernandez et al. 2016	No departure from the trend set by B	509	25	**21**
					Dry Mass	NS					
					Stem Length	NS					

Figure S1. Photos from the other two replicates of 'Red Sails' lettuce. In general, anthocyanin pigmentation was higher at a PPFD of 500 µmol m^{-2} s^{-1}, but pigmentation in the lower fractions of blue (10%) at the higher PPFD (500 µmol m^{-2} s^{-1}) looked visually similar to the pigmentation at the higher fraction of blue (30%) at the lower PPFD (200 µmol m^{-2} s^{-1}).

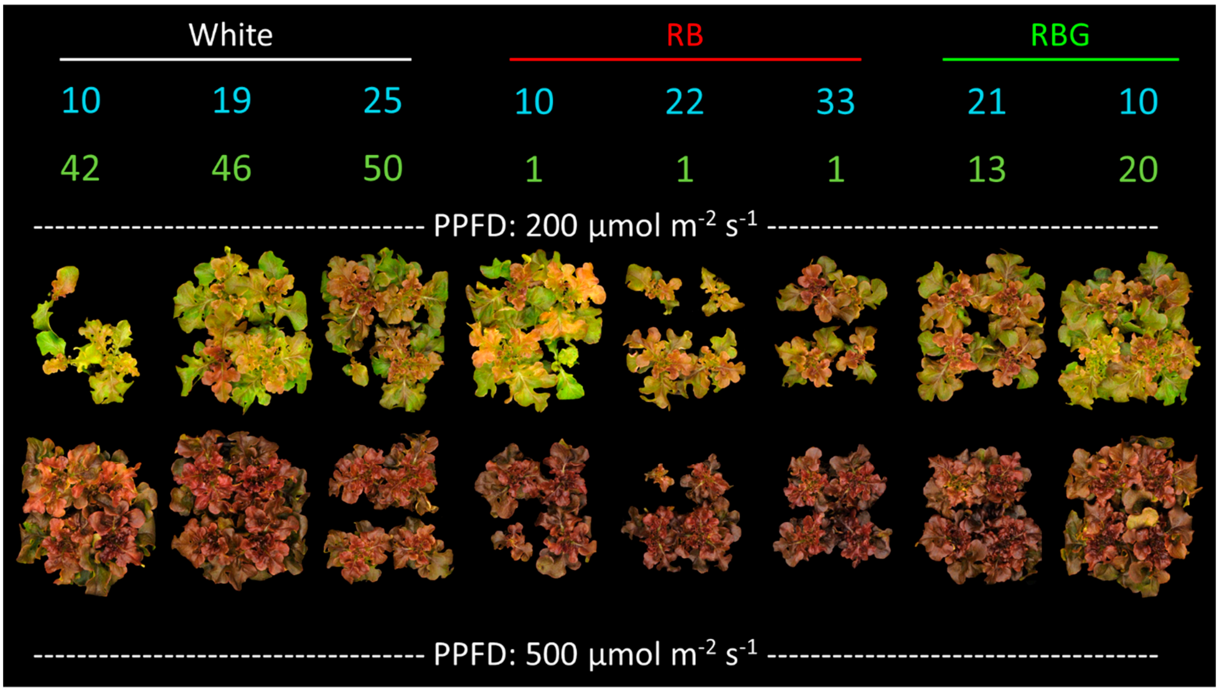

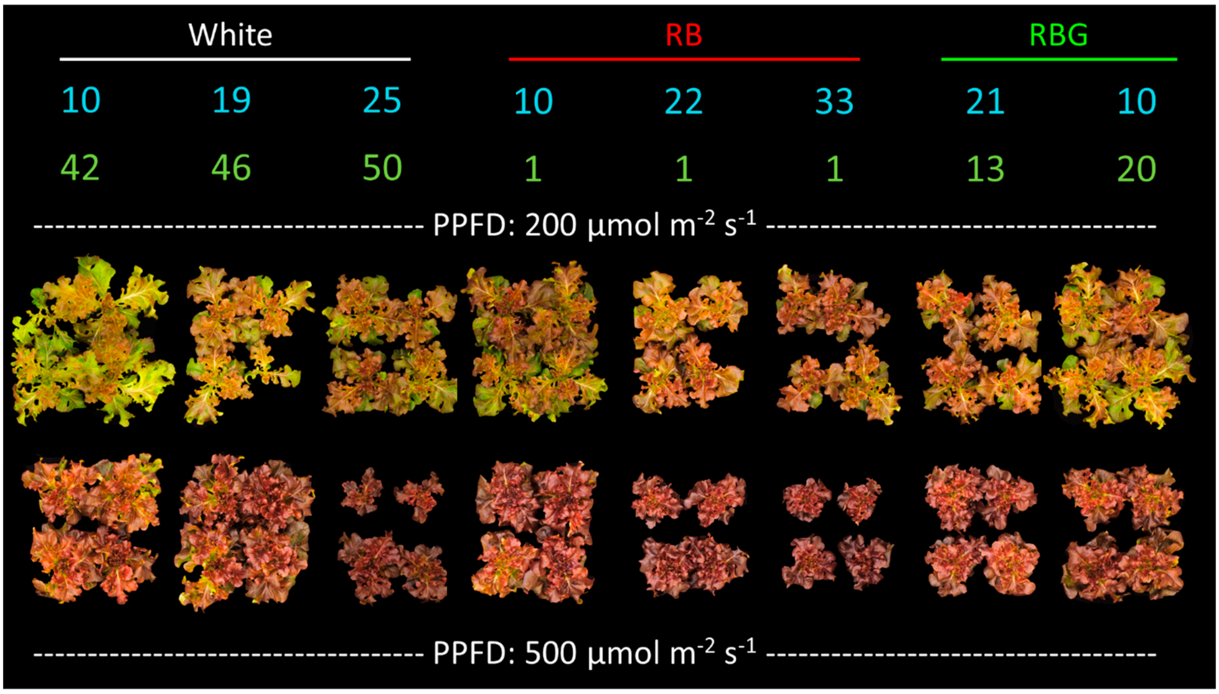

Supplement: Supplementary file 1 [file plants-10-00637-s001.pdf]
